# Effect of clone selection, nitrogen supply, leaf damage and mycorrhizal fungi on stilbene and emodin production in knotweed

**DOI:** 10.1186/1471-2229-11-98

**Published:** 2011-05-30

**Authors:** Marcela Kovářová, Tomáš Frantík, Helena Koblihová, Kristýna Bartůňková, Zora Nývltová, Miroslav Vosátka

**Affiliations:** 1 Institute of Botany, Czech Academy of Science, Průhonice 1, 252 43, Czech Republic; 2 VÚOS, Rybitví 296, 533 54 Rybitví, Czech Republic

**Keywords:** *Fallopia*, *F*. x*bohemica*, *F*. x*japonica*, *F*. x*sachalinensis*, *Polygonaceae*, *Reynoutria*, knotweed, emodin, stilbenes, piceid, resveratrol, leaf damage, mycorrhiza

## Abstract

**Background:**

*Fallopia japonica *and its hybrid, *F*. x*bohemica*, due to their fast spread, are famous as nature threats rather than blessings. Their fast growth rate, height, coverage, efficient nutrient translocation between tillers and organs and high phenolic production, may be perceived either as dangerous or beneficial features that bring about the elimination of native species or a life-supporting source. To the best of our knowledge, there have not been any studies aimed at increasing the targeted production of medically desired compounds by these remarkable plants. We designed a two-year pot experiment to determine the extent to which stilbene (resveratrol, piceatannol, resveratrolosid, piceid and astringins) and emodin contents of *F. japonica*, *F. sachalinensis *and two selected *F*. x*bohemica *clones are affected by soil nitrogen (N) supply, leaf damage and mycorrhizal inoculation.

**Results:**

1) Knotweeds are able to grow on substrates with extremely low nitrogen content and have a high efficiency of N translocation. The fast-spreading hybrid clones store less N in their rhizomes than the parental species. 2) The highest concentrations of stilbenes were found in the belowground biomass of *F. japonica*. However, because of the high belowground biomass of one clone of *F*. x*bohemica*, this hybrid produced more stilbenes per plant than *F. japonica*. 3) Leaf damage increased the resveratrol and emodin contents in the belowground biomass of the non-inoculated knotweed plants. 4) Although knotweed is supposed to be a non-mycorrhizal species, its roots are able to host the fungi. Inoculation with mycorrhizal fungi resulted in up to 2% root colonisation. 5) Both leaf damage and inoculation with mycorrhizal fungi elicited an increase of the piceid (resveratrol-glucoside) content in the belowground biomass of *F. japonica*. However, the mycorrhizal fungi only elicited this response in the absence of leaf damage. Because the leaf damage suppressed the effect of the root fungi, the effect of leaf damage prevailed over the effect of the mycorrhizal fungi on the piceid content in the belowground biomass.

**Conclusions:**

Two widely spread knotweed species, *F. japonica *and *F*. x*bohemica*, are promising sources of compounds that may have a positive impact on human health. The content of some of the target compounds in the plant tissues can be significantly altered by the cultivation conditions including stress imposed on the plants, inoculation with mycorrhizal fungi and selection of the appropriate plant clone.

## Background

In the Czech Republic, the genus *Fallopia *Adans. (*Polygonaceae*), also reported as a separate genus *Reynoutria *(Houtt.) Ronse Decr. consists of two species - *F. japonica *(Houtt.) Ronse Decr. (Japanese knotweed) and *F. sachalinensis *(F. Schmidt Petrop.) Ronse Decr. (Giant knotweed), and their hybrid, *F*. x*bohemica *(Chrtek et Chrtková) J. P. Bailey. The hybrid appeared when the two parental species, introduced into Europe from Asia in the 19^th ^century [1] as garden ornamentals, came into contact [1,2]. These perennial herbs are highly invasive, exotic species and recognized as a major environmental management problem in Europe [3,4] including Czech Republic [5]. However they also produce nectar and a plethora of organic substances that may be harvested for medicinal use [6]. Their use has not been only as melliferous or medical, but also as energetic plants (gross heating value comparable to that of wood, 18.4 GJ.t^-1^) with high growth rate and biomass production [7]. The knotweeds are utilized as a cultivated crop under rigid regulations in the Czech Republic [7,8]. Knotweeds are also used for soil amelioration, sewage treatment (because of its ability to accumulate heavy metals, especially Cd and Pb) and riverbank and sand hill reinforcement [7]. However, these qualities also contribute to its competitive advantage over other plants and result in monospecific stands, which are undesirable in nature reserves. There have been attempts to eradicate it by use of a glyphosate herbicide, combined with physical removal of the plants including sheep grazing, which was most efficient http://www.pod.cz/projekty/Moravka-kridlatka/Zaklnformace/metodikarev2602.pdf Herbicide treatment is, however, questionable as glyphosates contain phosphorus and may act as fertilizers enhancing knotweed growth especially on phosphorus-deficient soils.

Knotweed species differ in their clonal architecture, morphological and ecological properties. *F*. x*bohemica *has a high regeneration potential and a number of clones of the hybrid can be considered as the most successful representatives of the genus in terms of growth rate, regeneration and the establishment of new shoots. The species *F. sachalinensis *has the lowest regeneration ability [2,9]. *Fallopia *spp. also differ in their relative abundance in the Czech landscape [1], the hybrid is most widespread.

Knotweeds grow as pioneer species on volcanic soils [10-12] and coal ashes produced by power plants. Therefore, because of the very low N content in these substrates, they may be suitable for testing the effect of nitrogen content on the production of stilbenes (resveratrol) and emodin used in the pharmaceutical and food industries. There is evidence that secondary metabolites are produced in greater amounts in plants growing in low-nitrogen soils, because phenylalanine formed by photosynthesis is converted into phenolics under low N conditions [13]. However, under high N conditions phenylalanine is assimilated into proteins [14]. For these reasons, we selected ash as a model substrate in this experiment.

The pharmaceutical uses for knotweed have focused on stilbenes (resveratrol, piceatannol and their glucosides, piceid, resveratrolosid and astringins) and emodin. Resveratrol-glucosides (e.g., piceid) can be split into glucose and resveratrol, which increases the resveratrol levels. Therefore, we monitored the full range of resveratrol-containing substances, besides emodin.

Emodin is a biologically active, naturally occurring anthraquinone derivative (1,3,8-trihydroxy-6-methylanthraquinone) that is produced by lichens, fungi and higher plants that possess purgative, anti-inflammatory and anticancer effects [15-18]. In addition, emodin has been shown to induce apoptosis [19]. Resveratrol (3,4',5-trihydroxystilbene; molecular weight 228.2 g/mol) is a naturally occurring polyphenol that is present in various fruits and vegetables in significant levels. It has been shown to have antibacterial [20,21], antifungal [22], antioxidant, antimutagenic, anti-inflammatory, chemopreventive [23,24] and anticancer effects [25-27] including the inhibition of breast cancer [28]. It also inhibits α-glucosidase which is promising for the control of diabetes [29]. Knotweed is traditionally used for the production of resveratrol in Asia, particularly in China. In Europe, wine is the main source of this substance. A variety of stilbenes have been found in wine, including astringin, cis- and trans-piceid and cis- and trans-resveratrol.

Fungi (*Botrytis cinerea*) have been reported to increase the resveratrol content in wine grapes or in the leaves as a possible plant response to stress [24,30,31]. Resveratrol has antifungal activity and can restrict growth of *Trichosporon beigelii*, *Candida albicans *[22], *Penicillium expansum*, *Aspergillus niger *[32] and *A. carbonarius *[33]. Specifically it was found that 90 μg.ml^-1 ^of resveratrol reduced mycelial growth and the germination of *B. cinerea *conidia by 50% [34].

Some plants are known to possess advantageous features, such as mycorrhizal symbiosis, that enable them to overcome the challenges in their environment in harsh conditions. However, some plants react to the same mycorrhizal fungi adversely - namely plants that do not host mycorrhizal fungi, including all of the members of the family *Polygonaceae*, such as *Fallopia *[35]. Although knotweed is supposed to be a non-mycorrhizal plant, an arbuscular type of mycorrhiza was found in the roots of knotweeds growing in the volcanic soils of Mt. Fuji, Japan [12]. In addition, we found mycorrhizal colonisation in the roots of knotweeds sampled from a flooded alder forest in Moravia (Rydlová, personal communication). Therefore, mycorrhizal fungi may associate with knotweeds and potentionally alter their growth characteristics, their genotype and accumulation of plant secondary compounds [36]. Synthesis of resveratrol and its derivatives, especially piceid, is regulated by stilbene synthase (STS) gene which typically occurs in grapevines [37,38], wherefrom it was also transduced into different crop plants with the aim to increase their resistance against pathogens. STS gene is a typical stress-inducible/responsive gene. Fungi, not only pathogens but also mycorrhizal ones, belong to the stressors capable of induction of such responses in plant cells like chromatin decondensation enabling, besides others, gene expression [39]. It is thus to be expected that mycorrhizal colonization of knotweed roots may also induce STS gene expression in this plant, resulting in synthesis of resveratrol and its derivatives, namely piceid [40]. We thus chose to inoculate knotweeds with mycorrhizal fungi (a mixture of *Glomus *species) as a factor expected to increase the yield of these economically valuable compounds.

It has been reported that simulating herbivore (insect) grazing can increase the production of phenolic compounds in these plants [41]. Therefore, we exposed the knotweed plants to leaf damage to investigate if they would respond by increasing the production of stilbenes and emodin. In addition to studying the potential of traditional source of resveratrol in *Fallopia japonica*, we also wanted to study the "inland" sources of resveratrol and other stilbenes in *F*. x*bohemica*, along with the other parental species, *F. sachalinensis*. The resveratrol and piceid contents in these plants, in terms of dry mass, have not been discussed in the current literature. This study constitutes a novel contribution to the production of stilbenes and emodin in knotweeds. We use the term stilbenes for the sum of resveratrol and resveratrol contained in all its derivatives measured (piceatannol, piceid, resveratrolosid and astringins).

It can be expected that related taxa may respond differently to the same conditions. The present study compared the responses of two clones of the hybrid, along with its parental species. The following questions were addressed:

(1) How do the different species and clones of knotweed respond to soil nitrogen contents, in terms of stilbene and emodin production? (2) What is the effect of mycorrhizal inoculation/colonisation? (3) What is the effect of leaf damage to the individual species/clones on the production of stilbenes and emodin?

## Results

The biomass and chemical characteristics measured and tested by ANOVA are shown in Table 1. F-values and degrees of freedom may be found in Table S3 in Additional file 1. Only the three clones (FJ, FBM and FBP; for symbols see Methods) that contained stilbenes and emodin in higher concentrations were analysed for organic substances.

**Table 1:** Plant characteristics measured and tested in 2006 and 2007.

Experimental factors and their effect on plant characteristics - significance levels															
**Plant characteristics**		**Significance of factors and their interactions**													

	**year**	**A**	**B**	**C**	**D**	A*B	A*C	A*D	B*C	B*D	C*D	A*B*C	A*C*D	B*C*D	A*B*C*D

**Aboveground**		**CLONE**	**INOC**	**N**	**LF DMG**										

Plant d.m. (g)	06, 07	0.001	NS	0.001	NS	NS	NS	NS	NS	NS	NS	NS	NS	NS	NS

Plant height (cm)	06, 07	0.001	NS	0.001	NS	NS	NS	NS	NS	NS	NS	NS	NS	NS	NS

Stem no	06, 07	0.001	NS	0.001	NS	NS	NS	NS	NS	NS	NS	NS	0.05	NS	NS

Branch no	06, 07	0.001	NS	0.001	NS	NS	0.001	NS	NS	NS	0.05	NS	NS	NS	NS

Branch total length (cm)	2006	x	x	x	x	x	x	x	x	x	x	x	x	x	x

Leaf no	06, 07	0.001	NS	0.001	NS	NS	NS	NS	NS	NS	0.01	NS	NS	NS	NS

Stem water content (%)	06, 07	0.001	NS	NS	NS	NS	NS	NS	NS	0.05	NS	NS	NS	NS	NS

Leaf water content (%)	06, 07	0.001	NS	0.001	0.05	NS	NS	NS	NS	NS	0.05	NS	NS	NS	NS

Leaf area (cm2)	06, 07	0.001	NS	0.001	NS	NS	NS	NS	NS	NS	NS	NS	NS	NS	NS

SLA (cm2/g)	06, 07	NS	NS	0.01	NS	NS	NS	NS	NS	NS	NS	NS	NS	NS	NS

**Belowground**															

Root and rhizome d.m. (g)	2007	0.001	NS	0.001	0.05	NS	0.01	0.01	NS	NS	NS	NS	NS	NS	NS

N (%)	2007	0.001	0.001	0.001	0.001	0.001	0.001	0.001	NS	NS	NS	0.05	NS	NS	NS

C (%)	2007	NS	NS	NS	NS	NS	NS	NS	NS	NS	NS	NS	NS	NS	NS

Resveratrol (mass %)	2007	0.001	NS	NS	NS	NS	NS	NS	NS	NS	NS	NS	NS	NS	x

Piceid (mass %)	2007	0.001	NS	NS	NS	NS	NS	NS	NS	NS	NS	NS	NS	NS	x

Stilbenes (mass %)	2007	0.001	NS	NS	NS	NS	NS	NS	NS	NS	NS	NS	NS	NS	x

Emodin (mass %)	2007	0.001	NS	0.01	NS	NS	NS	NS	NS	NS	NS	0.05	0.05	NS	x

Infection rate M (%)	2007	0.001	x	NS	0.05	x	NS	0.01	x	x	NS	x	NS	x	x

Infection rate F (%)	2007	0.001	x	NS	0.05	x	NS	NS	x	x	NS	x	NS	x	x

Results of four-way ANOVA with the following factors: CLONE = knotweed clone; INOC = mycorrhizal inoculation; N = nitrogen level; LF DMG = leaf damage. Shown for data from 2007.

x = non-tested, NS = non-significant

### Differences between clones at two nitrogen levels

#### Biomass

**Figure 1:**
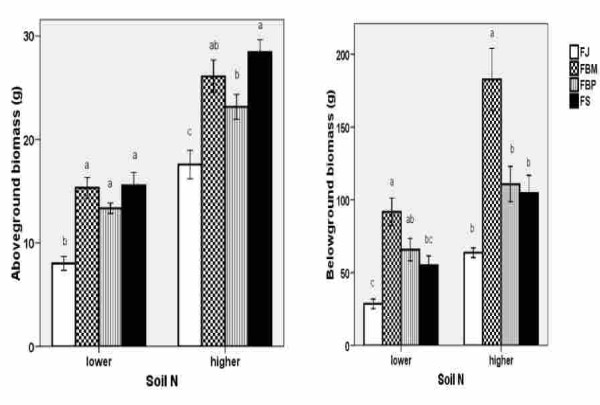
**Above- and belowground biomass of knotweed**. The above- (left) and belowground (right) biomasses (± S.E) of the control plants of the four knotweed clones at the two soil N levels in 2007. FJ = *Fallopia japonica*, FBM = *Fallopia *x*bohemica *from Mošnov, FBP = *Fallopia *x*bohemica *from Průhonice, FS = *Fallopia sachalinensis*. For both soil N levels, the same letters indicate non-significant differences, n = 10.

The aboveground biomasses (Figure 1a) of the clones differed and the pattern of the values was constant under lower and higher soil N levels in 2007. The lowest aboveground biomass was produced by FJ, followed by FBP. FBM and FS produced the highest biomass. Similar differences between the clones were measured in 2006 as well. FJ and FS produced the lowest belowground biomass, whereas FBM produced the highest biomass at both soil N levels (Figure 1b). As expected, the higher soil nitrogen supply increased the biomass of all of the clones.

#### Mycorrhizal colonisation

**Figure 2:**
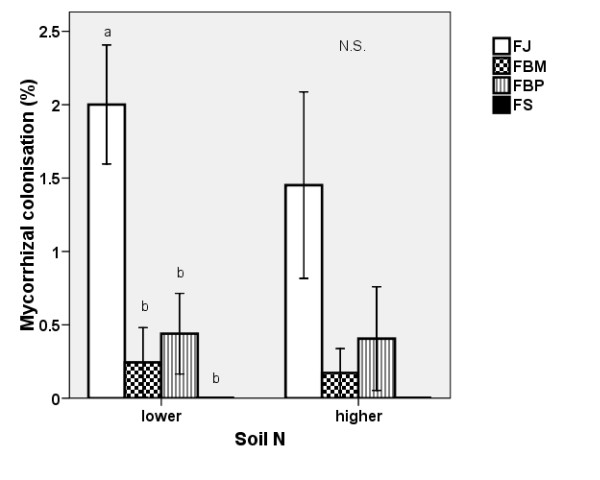
**Mycorrhizal colonisation of knotweed**. Mycorrhizal colonisation M (± S.E) in the inoculated plants of the four clones not exposed to leaf damage at the two soil N levels in 2007. FJ = *Fallopia japonica*, FBM = *Fallopia *x*bohemica *from Mošnov, FBP = *Fallopia *x*bohemica *from Průhonice, FS = *Fallopia sachalinensis*. For both soil N levels, the same letters indicate non-significant (NS) differences, n = 6.

No colonisation by mycorrhizal fungi was found in the roots of the non-inoculated plants. In the inoculated plants, vesicles and internal hyphae were present in the roots; however, arbuscules were not. Figure 2 shows that the inoculated plants developed very low intensity of mycorrhizal colonisation (M). FS had the lowest M value (with no mycorrhizal colonisation), whereas FJ had the highest M value and was the best host for the mycorrhizal fungi. The M values for the two hybrid clones fell in between the parents. The effect of nitrogen on mycorrhizal colonisation was not significant. The trend of the frequency of mycorrhizal colonisation (F) was similar to that of the M values and is not shown here.

#### Nitrogen Content in Plant Biomass

**Figure 3:**
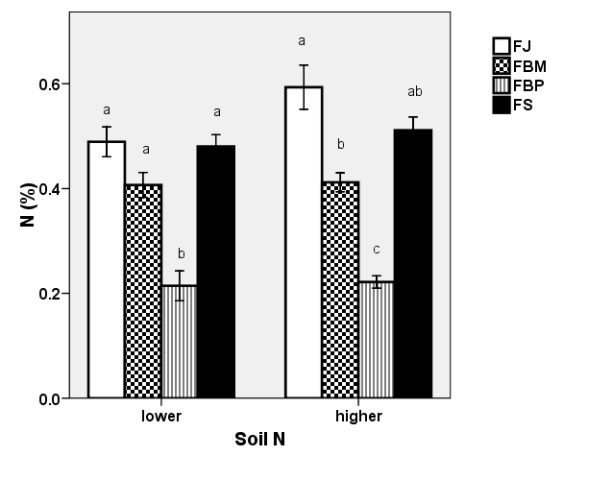
**Nitrogen content in knotweed rhizomes**. Nitrogen contents (± S.E) in the belowground biomass of control plants of the four clones at the two soil N levels in 2007. FJ = *Fallopia japonica*, FBM = *Fallopia *x*bohemica *from Mošnov, FBP = *Fallopia *x*bohemica *from Průhonice, FS = *Fallopia sachalinensis*. For both soil N levels, the same letters indicate non-significant differences, n = 6.

When the data for all the clones were combined, the higher soil N level was reflected in the higher N content of the belowground biomass (Table 1). However, the individual clones did not show a statistically significant increase between the lower and higher N levels (Figure 3).

There were differences in the N content of the belowground biomass at the two levels of soil nitrogen content studied between the particular clones. The two parental species had higher N contents than the hybrid clones. FBP had an extremely low nitrogen content of around 0.2% N.

#### Stilbene Content

**Figure 4:**
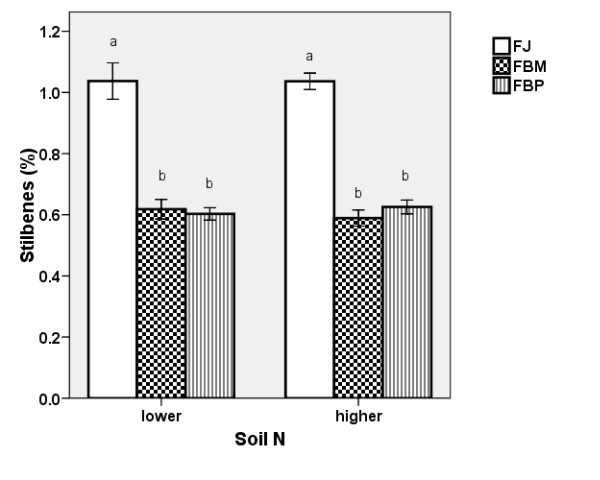
**Stilbene content in knotweed rhizomes**. Stilbene contents (± S.E) in the belowground biomass of the control plants of three clones at the two soil N levels in 2007, expressed as resveratrol including resveratrol contained in all its derivatives measured. FJ = *Fallopia japonica*, FBM = *Fallopia *x*bohemica *from Mošnov, FBP = *Fallopia *x*bohemica *from Průhonice. For both soil N levels, the same letters indicate non-significant differences, n = 6.

**Figure 5:**
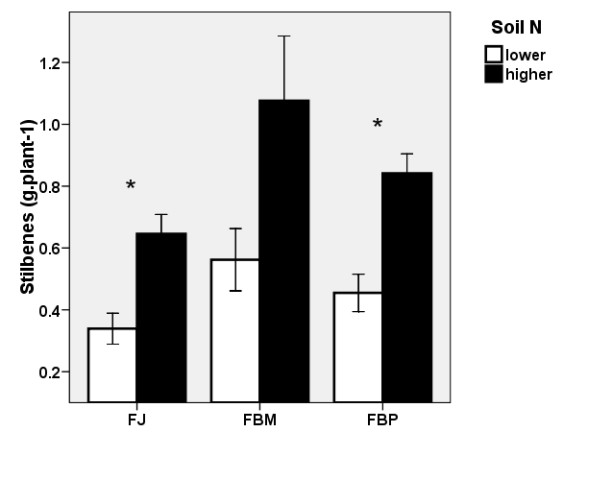
**Nitrogen effect on stilbene production in knotweed**. Effect of soil N level on the production of stilbenes per plant (± S.E) in the belowground biomass of the control plants of three clones in 2007. FJ = *Fallopia japonica*, FBM = *Fallopia *x*bohemica *from Mošnov, FBP = *Fallopia *x*bohemica *from Průhonice. Asterisks indicate significant differences, n = 6.

FJ had a higher stilbene content compared to the two *F*. x*bohemica *clones measured (Figure 4). Stilbene content was not affected by the soil N levels. However, the increase in the belowground biomass at the higher soil N level also brought about an increase in stilbene production (i.e., the amount of stilbenes in the belowground biomass of one plant). FBM had the highest stilbene production (Figure 5). The biomass increase as a result of N fertilisation did not restrict the production of stilbenes at the N levels used in our experiment.

#### Emodin content

**Figure 6:**
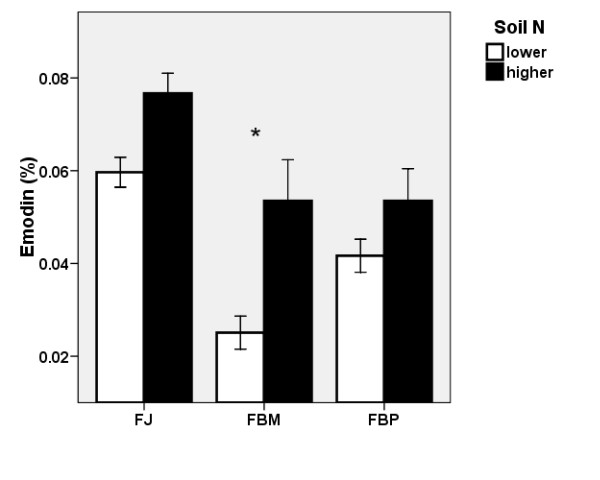
**Nitrogen effect on emodin content in knotweed**. Effect of the soil N level on the emodin content (± S.E) in the belowground biomass of the control plants of three clones in 2007. FJ = *Fallopia japonica*, FBM = *Fallopia *x*bohemica *from Mošnov, FBP = *Fallopia *x*bohemica *from Průhonice. Asterisks indicate significant differences, n = 6.

Figure 6 and Table 1 indicate that nitrogen had a positive effect on the emodin content in the belowground biomass of the knotweed. However, the increase of emodin content at higher soil nitrogen was only significant in FBM. The observed differences in emodin content of the individual clones were significant only at the lower soil N level, at which FJ produced the highest amount of emodin and FBM produced the lowest amount of emodin.

#### Effect of mycorrhizal inoculation

**Figure 7:**
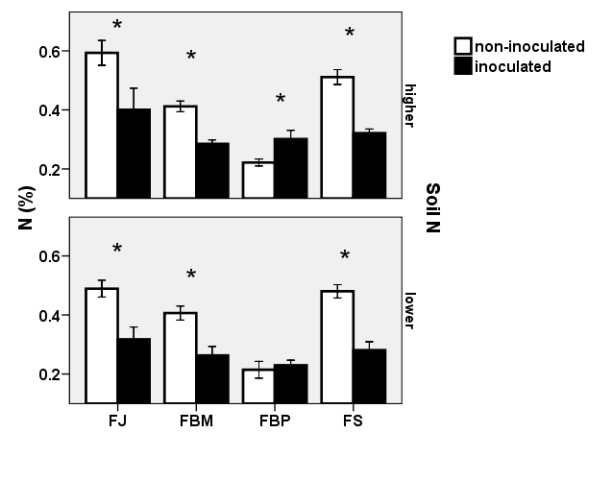
**Effect of mycorrhizal inoculation on nitrogen content in knotweed rhizomes**. Effect of mycorrhizal inoculation on the N content (± S.E) in the belowground biomass of four clones at the higher (top) and lower (bottom) soil N levels. Only plants without exposure to leaf damage in 2007 are shown. FJ = *Fallopia japonica*, FBM = *Fallopia *x*bohemica *from Mošnov, FBP = *Fallopia *x*bohemica *from Průhonice, FS = *Fallopia sachalinensis*. Asterisks indicate significant differences, n = 6.

Mycorrhizal inoculation significantly lowered the N content in the belowground biomass of all knotweed clones with the exception of FBP. This effect was observed to various degrees within the different clones (see the significant interaction between mycorrhizal inoculation, clone and nitrogen level - Table 1), most likely as a result of the competition the microbial community brought into the system with the inoculum. Figure 7 gives about a summary of the effect of mycorrhizal inoculation on the N content with different combinations of clones and soil nitrogen level. Mycorrhizal inoculation had no effect on the production and the concentration of resveratrol, stilbenes and emodin.

#### Effect of leaf damage

**Figure 8:**
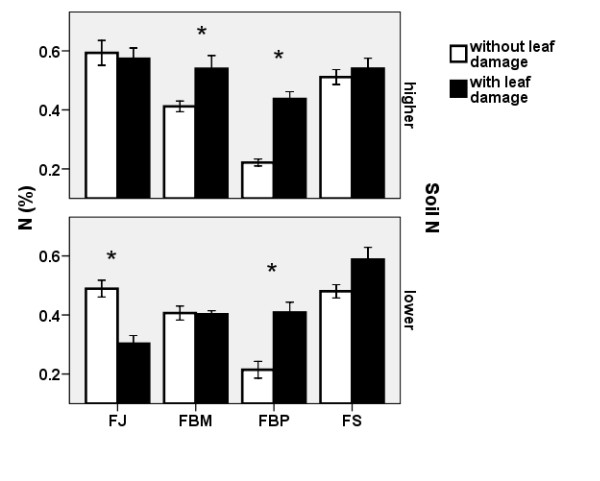
**Effect of leaf damage on nitrogen content in knotweed rhizomes**. Effect of leaf damage on the N content (± S.E) in the belowground biomass of four clones at the higher (top) and lower (bottom) soil N levels. Only non-inoculated plants in 2007 are shown. FJ = *Fallopia japonica*, FBM = *Fallopia *x*bohemica *from Mošnov, FBP = *Fallopia *x*bohemica *from Průhonice, FS = *Fallopia sachalinensis*. Asterisks indicate significant differences, n = 6.

The leaf damage negatively affected leaf water content, mycorrhizal colonisation and belowground biomass (Table 1). However, leaf damage had no effect on aboveground biomass, leaf area and SLA. The effect of leaf damage on the N content was more complicated (see Table 1, significant interactions). Leaf damage increased the N content in FBP at both soil N levels and in FBM at the higher soil N level and decreased the N content in FJ at the lower soil N level (Figure 8). Leaf damage had no effect on the N content in the belowground biomass of the knotweed in the inoculated variants.

Even though the effect of leaf damage on resveratrol and emodin content was not significant at P = 0.05 (Table 1), leaf damage significantly increased the resveratrol (from 0.027% to 0.035%) and emodin (from 0.052% to 0.062%) content in belowground biomass of the non-inoculated knotweed plants. Leaf damage had no effect on stilbene content but enhanced piceid content in the inoculated *F. japonica *(from 0.93% to 1.13%). The leaf damage significantly lowered the intensity of mycorrhizal colonisation (both F and M - Table 1). M value decreased from 1.7% to 0.6% in FJ in response to leaf damage.

For more results see Additional file 1.

## Discussion

Even though resveratrol is produced commercially from the Japanese knotweed in Asia, there is little knowledge concerning resveratrol and piceid contents of knotweed clones within the scientific literature. The lack of information may be due to the various efficiencies of the variety of extraction agents used or due to the measurement of the extract rather than the whole plant. We measured the stilbene yields of these plants under specific conditions designed to increase stilbene production by the knotweed. In addition, we determined the most efficient clone for the production of resveratrol and piceid.

### Seasonal variability in the resveratrol and piceid contents

**Figure 9:**
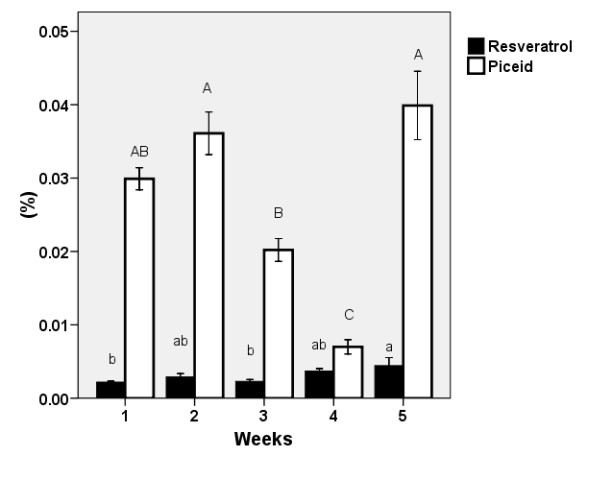
**Seasonal variation of stilbene content in knotweed leaves**. Seasonal variation in the content of resveratrol and piceid (± S.E) in overall foliage per stem from the semi-natural population of *F. japonica*, from April 27 (plants ca 1 m high) to May 24 (fully grown plants), 2007. The same letters indicate non-significant differences in resveratrol (lower case) and piceid (upper case) contents, n = 10.

Although it may be more economical to process the aboveground biomass rather than the rhizomes and roots, belowground biomass has a much higher content of stilbenes and emodin. Additionally, we found (unpublished data) that stilbene content in rhizomes peaked at the end of the growing season. Supposed that there is transport of these substances to the shoots in the spring, a seasonal variation may be then expected. A pronounced seasonal variation in resveratrol and piceid contents occurred in the aboveground biomass of the *F. japonica *at the beginning of its growth cycle (Figure 9). Knotweed is known for its fast growth rate in the spring and can produce up to 100 mm a day. Thus the transport of substances from the rhizomes to the shoots results in a dillution in the total biomass pool. Both resveratrol and piceid possess antifungal activities and are present in high concentrations in the rhizomes (0.04% and 1%, resp.); when transported into shoots, they help to protect the fresh tissues from pathogens. In the foliage, the concentration of resveratrol gradually increased up to 0.005%. The concentration of piceid in the aboveground biomass showed high initial values that were followed by a significnat decrease before the full development of the shoots, and a subsequent increase up to 0.04%. It is reasonable to assume that the transition between resveratrol (an aglycon) and piceid (a glucoside) depends on the amount of available glucose produced during photosynthesis.

### Interaction of leaf damage, mycorrhizal colonisation and piceid in *F. japonica*

Hartley and Firn [42] found increased levels of phenolics in damaged birch leaves. Similarly, increased concentrations of some phenolics including resveratrol in wounded spruce trees have been detected [43]. In our experiment, leaf damage elicited a positive effect on the piceid content in *F. japonica*, which is in line with these studies. *F. japonica *was most substantially affected by leaf damage out of the clones, most likely because it had the highest content of resveratrol derivatives, the majority of which was piceid (resveratrol-glucoside). Piceid may be viewed as a source from which resveratrol may be generated and has been shown to exert fungistatic effects; resveratrol itself was present in knotweed at very low amounts.

**Figure 10:**
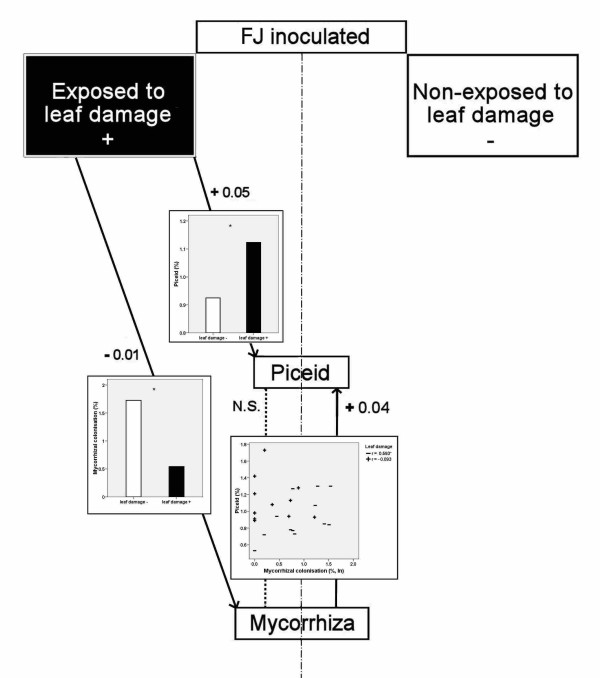
**Leaf damage, piceid and mycorrhiza in knotweed**. Relationships in the inoculated FJ between leaf damage (treatment, left side; control, right side), piceid and mycorrhiza. The two N levels are combined. Significant relationships, full line (level of significance indicated); non-significant relationships, dashed line (N.S.), n = 12. Minus, inverse proportionality; plus, direct proportionality. For more information, please see the text.

The most interesting findings pertain to the relationship between piceid, leaf damage and the intensity of mycorrhizal colonisation. In inoculated *F. japonica*, leaf damage increased piceid content, decreased the intensity of mycorrhizal colonisation and weakened the relationship between piceid and the intensity of mycorrhizal colonisation, which was significant and positive in plants not exposed to leaf damage. In plants exposed to leaf damage, no correlation was found between the intensity of mycorrhizal colonisation and piceid content in the belowground biomass of *F. japonica *because leaf damage increased its piceid content. However, there was a significant correlation in the undamaged plants. Figure 10 summarises these results, which suggest that in the Japanese knotweed, leaf damage stimulates piceid production to a greater extent than colonisation by mycorrhizal fungi. Leaf damage may even control the intensity of knotweed mycorrhizal colonisation, presumably because of the increased production of piceid.

Despite the fact that the mycorrhizal colonisation of the knotweed roots was low (2%), a significant effect of mycorrhizae on the piceid levels in plants not exposed to leaf damage was still observed. Recent research on mycorrhiza has devoted more attention to the effects of low levels of colonisation by mycorrhizal fungi on their plant hosts [44,45]. Knotweeds are non-obligately-mycorrhizal plants and maintain low colonisation levels when they are grown in monocultures. However, when grown together with a typical mycorrhizal plant, such as leguminous melilot, they can be colonised up to 60% [8]. Our findings may be a small contribution to this discussion which touches upon new paradigms in mycorrhizal science.

Piceid is at least as effective in the prevention of fungal penetration into leaves as resveratrol. It was found that sorghum seedlings infected with the anthracnose pathogen *Colletotrichum sublineolum *accumulated trans-piceid as the major stilbene metabolite, along with an unknown resveratrol derivative [46]. In vitro experiments [47] revealed that piceid and resveratrol had an inhibitory effect on the germination of the phytopathogenic fungus *Venturia inaequalis *and its penetration through the cuticular membrane, which improved the resistance of plant leaves. It has been reported that resveratrol can be transformed into piceid by *Bacillus cereus *[48]. This evidence suggests that these two closely related substances have similar antifungal effects and can create an efficient barrier against the penetration of pathogenic fungi. In the sorghum cultivars [46], piceid was induced 48 hours after mycorrhizal inoculation. This result agrees with our finding that the exposure of knotweed leaves to leaf damage, as well as mycorrhizal colonisation of the knotweed roots, increased the piceid concentration in the belowground biomass. We hypothesise that damage to the leaves increased the piceid level, which then restricted the mycorrhizal colonisation of the roots.

### Piceid/N ratio

**Figure 11:**
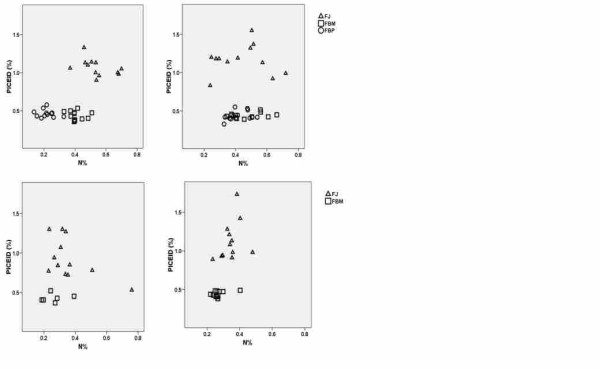
**Piceid and nitrogen in rhizomes of differently treated knotweed plants**. Relationships between piceid and nitrogen in the belowground biomass of the control plants (a), damaged plants (b), inoculated plants (c) and inoculated and damaged plants (d) of the FJ, FBM and FBP clones and the two soil N levels combined, in 2007, n = 12.

As shown in Table 1, N content in rhizomes was strongly affected by all the factors tested in the pot experiment. We found an interesting relationship between N and piceid contents in rhizomes of knotweed clones. Piceid is a transient molecule and its content increases when there are enough energy-rich glucosides available; resveratrol is a suitable receptor on which glucosides are bound. According to the protein competition model of phenolic allocation [14], plants use photosynthetic carbon products (phenylalanine) predominantly for the synthesis of secondary metabolites, such as phenolics, alkaloids, stilbenes and/or lignin when the nitrogen availability is low and for the synthesis of proteins at higher N concentrations. A negative correlation between leaf phenolic and nitrogen contents has been reported [49]; however, we did not find a relationship between the nitrogen and piceid contents in the belowground biomass of the individual knotweed clones tested. Figure 11 shows the consistent differences between the piceid content of the clones related to the nitrogen content. The highest concentrations of piceid were measured in the belowground biomass of FJ. The two hybrid clones, FBM and FBP, had about the same piceid content but differed in their N content (Figure 11a). The exposure of these clones to leaf damage eliminated this difference by increasing the very low N content in FBP. The positive effect of both leaf damage and mycorrhizal inoculation on the ratio of piceid to N content is a novel finding.

In another experiment with *F*. x*bohemica *[8] we found that the piceid/N ratio significantly decreased (from 1.7 to 1.2) because of the presence of melilot, which enriched the system with nitrogen fixed by its rhizobia. In this experiment, the piceid/N ratio was significantly increased by leaf damage (Figure 11b) in FJ (from 2 to 3) and by mycorrhizal inoculation (Figure 11c) both in FJ (from 2 to 3) and FBM (from 1 to 1.7). Two things that likely contributed to the increased piceid/N ratio were the net increase of piceid in FJ subjected to leaf damage, resulting from a defence response, and a decrease of nitrogen in FJ and FBM, resulting from mycorrhizal inoculation. This decrease was likely caused by competition with soil microorganisms for nitrogen.

## Conclusions

Significant production of stilbenes and emodin was found in two widely spread knotweeds, *F. japonica *and *F*. x*bohemica*, which were cultivated in pots in the ash substrate. The content of some target compounds in the plant tissue can be significantly altered by these means:

1) manipulation of the nitrogen content in the substrate - the increase in biomass as a result of the N fertilisation did not restrict the production of stilbenes at the N levels used in our experiment;

2) imposing stress on plants - leaf damage increased the resveratrol and emodin contents in the belowground biomass of the non-inoculated knotweed plants;

3) inoculation with mycorrhizal fungi - mycorrhizal fungi elicited an increase in the piceid (resveratrol-glucoside) content in the belowground biomass of *F*. japonica, but only in the absence of leaf damage.

4) selection of the appropriate plant clone - the production of secondary compounds differed among the plant clones tested. Despite the higher concentration of these substances in *F. japonica*, their total production is higher in the two clones of *F*. x*bohemica*, because of their higher biomass produced per plant.

Both *Fallopia japonica *and the two clones of *F*. x*bohemica *(FBM and FBP) are promising sources of resveratrol and piceid, which possess the potential to protect and improve human health.

## Methods

### Plant material

Prior to the pot experiment, a survey was made concerning the resveratrol and piceid contents using a collection of genetically defined clones with known ploidy levels, in the experimental garden of the Institute of Botany, Czech Academy of Science [50]. Rhizomes were sampled from 20 different clones including *Fallopia japonica*, *F. sachalinensis *and *F. xbohemica*. *F. japonica *occurs only as a singular, octoploid clone, whereas *F. sachalinensis *and F. x*bohemica *were found as tetraploid, hexaploid and octoploid clones. As there was no relationship between the ploidy level and the content of either resveratrol and/or piceid in the knotweed rhizomes, the choice of which hybrid clones to use in our pot experiment (FBM and FBP) was made by using the following criteria: (1) resveratrol and piceid content, (2) environmental safety (some of these clones were appointed as "extremely dangerous" and it was recommended that we would avoid those indicated as dangerous, and implement only clones which are safe enough to work within the pot experiment - traditionally known as non-spreading, i.e., occupying the same space in the long-term and forming no viable seeds; B. Mandák, personal communication), (3) reasonable growth (stable, persistent and vital populations) and (4) rhizome availability (sufficient amounts/proportion of young rhizomes; old populations were avoided). Out of the parental clones, *F. japonica *var. *japonica *(octoploid) was an obvious choice as the other clone of *F. japonica, F. japonica *var. *compacta *does not grow well. *F. sachalinensis *(tetraploid) was sampled from a population closest to the location where the hybrid was first described.

The pot experiment started in September 2005 when the rhizome segments of the knotweed were planted. They were ca. four cm long; we rid the plant rhizomes of all roots and immersed them for 15 min. into a 20% solution of Savo (sodium hypochlorite) for sterilisation of their surface. The rhizomes were sampled from four clones of *Fallopia*, namely *F. sachalinensis *(F. Schmidt Petrop.) Ronse Decr. (n = 44; genome size: 2C-values DNA = 4.345 pg; FS) from a wild stand in Průhonice on the banks of the Botič stream, *F*. x*bohemica *(Chrtek et Chrtková) J. P. Bailey (n = 66; genome size: 2C-values DNA = 6.918 pg; FBP) from a wild stand alongside the Botič stream, Průhonice, *R *xbohemica (n = 66; genome size: 2C-values DNA = 6.945 pg; FBM) from a field culture in Mošnov and *F. japonica *(Houtt.) Ronse Decr. (n = 88; genome size: 2C-values DNA = 9.541 pg; FJ) from a semi-natural population in an abandoned garden in Prague. The seasonal variation in the resveratrol and piceid content in the shoots was estimated in 2007. Genome size was estimated by the same method as described by [50]

### Experimental design

The pot volume was 10 l, with the bottoms covered by a sterile textile to keep the material inside; a total of 320 pots were used. Two thirds of the volume was filled with a 9:1 v/v mixture of ash from a granulating furnace (for chemical composition see Table 2) delivered directly from its source, the power station in Božkov near Plzeň, and sterile (25 kGrey gamma-irradiated) low-N bark compost (0.6-0.7% N, pH 5.5). One half of the pots was inoculated with a mixed inoculum of 3 isolates of arbuscular mycorrhizal (AM) fungi: *Glomus mossae *BEG95, *G. claroideum *BEG96 and *G. intraradices *BEG140 (all originated from man-made sites). The inoculum was produced on roots of maize grown in a mixture of zeolite and river sand (1:1 v/v) in the greenhouse for 4 months. Each inoculated pot received 100 ml of inoculum of AM fungi, consisting of equal volumes of spores, colonized root pieces and fragments of extraradical mycelium of each fungal isolate. The second half of the pots (non-inoculated control treatment) was supplied with the same quantity of heat-sterilised inoculum plus 100 ml of inoculum-filtrate to obtain a similar quantity of organic matter and microbial conditions (except viable AM fungi) in all treatments. The pots were filled to the rim with the same substrate and the surface was covered with 1 L of the same sterilised bark compost which was used in the mixture with ash. One hundred ml of filtrate from the bark compost containing native microflora (but not AM fungi) was added to all treatments. The filtrates from the inoculum and from the bark compost were prepared by shaking 100 g of inoculum or compost, respectively, with 1 L of deionized water for 30 min and filtered twice through filter paper with a pore size of 10 μm. The pots were then placed in a greenhouse for the winter but kept outside on the greenhouse tables during the growing season. A drip-irrigation system was applied (Rainbird, USA) with a separate tube for each pot, which prevented the sun from burning the wet leaves and cross-contamination between inoculated and non-inoculated substrates.

**Table 2:** Elemental content of the alcaline (pH(H_2_O) = 8.00; pH(KCl) = 7.94) ash used as a substrate in the pot experiment.

Chemical composition of the pot substrate	
**Element**	**Content (ppm)**

C-CO_3_	1 480

P	102

Ca	32 842

Mg	1 110

K	789

Ag	< 5.6

As	67

Ba	730

Cd	< 1.2

Co	24

Cr	46

Cu	240

Hg	0.9

Ni	35

Pb	5.8

Zn	52

Majority of its particles fell in the category 10-50 μm (50%), followed by 100-2000 μm (40%).

Equal amounts of phosphorus (90 mg/pot, equivalent of 20 kg/ha), in the form of KH_2_PO_4_, were applied to the pots at the beginning of the experiment and again in September 2006. All pots (area 452 cm^2^) were treated with 20 kg/ha of N in the form of carbamide (NH_2_-CO-NH_2_), which contained 46% N, in June 2006, and only the high-N plants received four additional N doses from August to September in 2006 and 2007. In the summer of 2007, when the leaves were fully developed, all of them were gently punctured with a sterilised stainless steel pet brush with a wire diameter of 0.25 mm and a density of 1242 pcs/dm^2^. The area of the brush was 75 × 28 mm; the leaves had a strip of 28 mm punctured across their width.

There were 10 replicates for each clone × N × mycorrhizal colonization × leaf damage combination, the pots were fully randomized.

### Plant growth analysis

The process of translocation of nutrients as well as secondary compounds into the underground organs ceases simultaneously with plant growth decline at the end of the growth season, typically in October. In the pots, the plants were harvested in October 2006 for aboveground biomass and in October 2007 for both above- and belowground biomass. On the day of sampling, the shoots were cut, and the following plant growth characteristics of each ramet were recorded: number of leaves, the leaf area (cm^2^), the fresh and dry weight of leaves and stems (g). Leaf area was measured using LI-COR LI-3000 planimeter.

Using these data, the aboveground biomass, leaf and stem water content (100*(fresh weight - dry)/fresh weight) and specific leaf area (SLA = leaf area/leaf dry wt; cm^2^.g^-1^) to assess leaf thickness were calculated. The belowground biomass was measured after washing and cleaning of the roots and rhizomes that were dried, weighed, ground and analysed for C, N and organic substances.

To estimate seasonal variability of resveratrol and piceid contents, leaves were sampled at the beginning of the growth season, weekly from April 27 to May 24, dried, ground and analysed for organic substances.

### Mycorrhizal colonisation assessment

The roots were washed from the soil on a sieve, cut into one to two cm segments and stained with 0.05% Trypan blue in lactoglycerol [51]. Mycorrhizal colonisation (arbuscules, vesicles and internal hyphae) was checked under a compound microscope (Olympus B × 41) at 100 × magnification. The frequency (F) and intensity (M) of mycorrhizal colonisation of the roots were evaluated according to previously described methods [52].

### Chemical analyses

Stilbenes, including resveratrol, piceatannol and resveratrol glucosides (piceid, resveratrolosid, astringins), were analysed as well as emodin. To determine the resveratrol content, it is important to measure not only the resveratrol content but also the content of the resveratrol glucosides. These are easily split into resveratrol and sugars, a process that will increase the measured resveratrol content. (A simple low-cost patented technological process for raising resveratrol content in knotweed includes fermentation for 24-96 h at 20-50°C; see http://en.cnki.com.cn/Article_en/CJFDTOTAL-JXHG200805014.htm) Dry and finely (0.01 mm sieve) ground samples were extracted with 60% ethanol, as it was the most efficient extractant for both resvertrol and its glucosides. The extracts were analysed by validated HPLC-UV method. Instrument: Shimadzu LC2010C HT with UV detection 306 nm; column: Phenomenex synergi Hydro-RP 80A, 250 × 4.6 mm, 4 μm (30°C); flow rate: 1.5 ml.min^-1^; mobile phases: A - 10 mM ammonium acetate at pH 4.15 using acetic acid, B - acetonitrile, concentration gradient from 7% to 90%. Standards: Piceid: 98%, OSA: 59394; Resveratrol: 99%, Sigma-Aldrich; Emodin: 98%, OSA: 59395; Astringin: 99%, Polyphenols; Piceatannol: 99%, Sigma Aldrich. External standard method with calibration curve was used for quantification.

Nitrogen and carbon in rhizomes were measured in the Analytical Laboratory of the Botanical Institute, Czech Acad. Sci., Průhonice, as total elemental content after combustion in an oxygen atmosphere (N, C) in a Carlo-Erba analyser. Macronutrients in the ash were measured according to [53] apart of phosphorus which was measured in bicarbonate extract at pH 8.5 [54]. Particle size analysis of the ash substrate was made in sedimentograph Analysette 20 by Fritsch. Analyses of heavy metals were bestowed by ash supplier Plzeňská teplárenská, a.s.

### Data analysis

Data with normal distributions were tested by multi-way ANOVA and Tukey test. Other data were evaluated using nonparametric tests such as Kruskal-Wallis and Mann-Whitney. Correlation analysis used Pearson's coefficient r. The statistical program employed was SPSS 14.0. The significance level of P = 0.05 was used if not otherwise indicated.

## Authors' contributions

MK conceived the study, coordinated the experiments and drafted the manuscript. TF performed the statistical analyses, prepared the graphs and commented on the draft text. KB performed the mycorrhizal part of the study. HK participated substantially in the experimental work and in the communication between the group members. ZN performed the organic chemical analyses. MV designed the experiment and contributed to the written manuscript. All authors read and approved the final paper.

## Supplementary Material

Additional file 1 **Supplementary data on pot experiment**. This file contains more details on statistics (F-values and degrees of freedom) and several other plant characteristics, such as stem, branch and leaf numbers, leaf area, SLA, stem and leaf water contents and carbon content, reflecting the effects of experimental factors. Click here for file
