# Supplementary material for: Effect of clone selection, nitrogen supply, leaf damage and mycorrhizal fungi on stilbene and emodin production in knotweed
Source: BMC Plant Biol. 2011 May 30;11:98. doi: 10.1186/1471-2229-11-98 (PMC3123627; doi:10.1186/1471-2229-11-98)
Supplement: Additional file 1 — Supplementary data on pot experiment. This file contains more details on statistics (F-values and degrees of freedom) and several other plant characteristics, such as stem, branch and leaf numbers, leaf area, SLA, stem and leaf water contents and carbon content, reflecting the effects of experimental factors. [file 1471-2229-11-98-S1.DOC]

**Additional file 1**

**Supplementary data on pot experiment**

Table 1 does not show all the information produced by four-way ANOVA analyses of the experimental data. Table 3 was added with F-values and degrees of freedom**.** The following are our comments to the measured plant characteristics that are either not included or only marginally mentioned in the Results section: FJ was taller than the other clones; the hybrid FBM clone had higher stem and leaf numbers than the other clones; the two parental species had higher branch numbers than the two hybrid clones; higher nitrogen level increased plant height, stem, branch and leaf numbers and leaf area (LA) but decreased leaf water content and SLA (only for all the clones together; not for any particular clone). FJ had a lower LA and higher stem water content than the remaining clones. Stress decreased leaf water content. Carbon content was very stable and unaffected by any of the experimental effects.

Experimental factors and their effect on plant characteristics – F- values

| **Plant characteristics** | **F-values and degrees of freedom of factors and their interactions tested for data from 2007** | | | | | | | | | | | | | |
| --- | --- | --- | --- | --- | --- | --- | --- | --- | --- | --- | --- | --- | --- | --- |
|  | **A** | **B** | **C** | **D** | **A*B** | **A*C** | **A*D** | **B*C** | **B*D** | **C*D** | **A*B*C** | **A*C*D** | **B*C*D** | **A*B*C*D** |
| **Aboveground** | **CLONE** | **INOC** | **N** | **LF DMG** |  |  |  |  |  |  |  |  |  |  |
| Plant dry mass (g) | 81.7 | 0.1 | 576.3 | 2.4 | 0.7 | 1.8 | 0.2 | 1.8 | 0.1 | 0.1 | 0.3 | 0.9 | 0.1 | 0.3 |
|  | 3 | 1 | 1 | 1 | 3 | 3 | 3 | 1 | 1 | 1 | 3 | 3 | 1 | 3 |
| Plant height (cm) | 128.9 | 0.1 | 121.1 | 0.3 | 1.5 | 0.5 | 0.1 | 0.2 | 1.8 | 1.5 | 0.6 | 0.7 | 2.0 | 2.5 |
|  | 3 | 1 | 1 | 1 | 3 | 3 | 3 | 1 | 1 | 1 | 3 | 3 | 1 | 3 |
| Stem no | 17.8 | 0.1 | 27.6 | 0.5 | 0.5 | 2.2 | 1.4 | 0.1 | 0.5 | 0.1 | 0.5 | 3.6 | 0.9 | 0.6 |
|  | 3 | 1 | 1 | 1 | 3 | 3 | 3 | 1 | 1 | 1 | 3 | 3 | 1 | 3 |
| Branch no | 10.6 | 0.1 | 56.3 | 1.4 | 0.4 | 9.4 | 0.1 | 1.9 | 0.2 | 5.8 | 0.9 | 0.9 | 0.5 | 0.6 |
|  | 3 | 1 | 1 | 1 | 3 | 3 | 3 | 1 | 1 | 1 | 3 | 3 | 1 | 3 |
| Leaf no | 26.4 | 2.7 | 278.8 | 1.7 | 1.4 | 0.3 | 0.4 | 0.5 | 0.1 | 9.6 | 1.7 | 0.9 | 1.9 | 0.7 |
|  | 3 | 1 | 1 | 1 | 3 | 3 | 3 | 1 | 1 | 1 | 3 | 3 | 1 | 3 |
| Stem water content (%) | 34.1 | 0.1 | 0.4 | 0.1 | 2.1 | 1.9 | 1.4 | 0.1 | 4.8 | 0.8 | 1.1 | 1.9 | 2.6 | 0.4 |
|  | 3 | 1 | 1 | 1 | 3 | 3 | 3 | 1 | 1 | 1 | 3 | 3 | 1 | 3 |
| Leaf water content (%) | 50.4 | 0.2 | 12.7 | 4.2 | 1.6 | 2.3 | 0.5 | 0.5 | 0.2 | 5.8 | 0.8 | 0.7 | 1.6 | 1.5 |
|  | 3 | 1 | 1 | 1 | 3 | 3 | 3 | 1 | 1 | 1 | 3 | 3 | 1 | 3 |
| Leaf area (cm2) | 74.2 | 0.1 | 495.7 | 1.9 | 0.6 | 2.6 | 0.6 | 1.5 | 0.1 | 0.1 | 0.6 | 0.3 | 0.4 | 0.3 |
|  | 3 | 1 | 1 | 1 | 3 | 3 | 3 | 1 | 1 | 1 | 3 | 3 | 1 | 3 |
| SLA (cm2/g) | 0.8 | 1.0 | 9.8 | 0.9 | 2.6 | 1.7 | 1.4 | 0.5 | 0.1 | 0.7 | 1.4 | 2.2 | 1.0 | 0.2 |
|  | 3 | 1 | 1 | 1 | 3 | 3 | 3 | 1 | 1 | 1 | 3 | 3 | 1 | 3 |
|  |  |  |  |  |  |  |  |  |  |  |  |  |  |  |
| **Belowground** |  |  |  |  |  |  |  |  |  |  |  |  |  |  |
| Root and rhizome dry mass (g) | 51.9 | 189.2 | 164.8 | 4.4 | 2.0 | 4.0 | 4.2 | 0.1 | 3.0 | 1.3 | 2.0 | 0.5 | 0.1 | 0.2 |
|  | 3 | 1 | 1 | 1 | 3 | 3 | 3 | 1 | 1 | 1 | 3 | 3 | 1 | 3 |
| N (%) | 26.4 | 161.6 | 30.2 | 13.1 | 14.6 | 5.4 | 13.7 | 0.6 | 3.2 | 1.5 | 3.2 | 1.95 | 2.6 | 2.1 |
|  | 3 | 1 | 1 | 1 | 3 | 3 | 3 | 1 | 1 | 1 | 3 | 3 | 1 | 3 |
| C (%) | 0.8 | 3.2 | 0.1 | 0.8 | 1.4 | 0.9 | 2.4 | 0.1 | 0.2 | 0.2 | 1.1 | 0.4 | 3.0 | 1.8 |
|  | 3 | 1 | 1 | 1 | 3 | 3 | 3 | 1 | 1 | 1 | 3 | 3 | 1 | 3 |
| Resveratrol (mass %) | 12.0 | 1.4 | 1.9 | 0.1 | 0.6 | 0.1 | 0.2 | 0.1 | 2.0 | 0.1 | 0.1 | 0.1 | 0.2 | x |
|  | 2 | 1 | 1 | 1 | 1 | 2 | 2 | 1 | 1 | 1 | 1 | 2 | 1 |  |
| Piceid (mass %) | 209.6 | 2.0 | 0.2 | 3.3 | 1.7 | 0.1 | 2.9 | 0.0 | 0.6 | 0.2 | 0.6 | 0.1 | 1.0 | x |
|  | 2 | 1 | 1 | 1 | 1 | 2 | 2 | 1 | 1 | 1 | 1 | 2 | 1 |  |
| Stilbenes (mass %) | 67.9 | 0.2 | 0.4 | 1.0 | 0.2 | 0.2 | 0.7 | 0.2 | 1.5 | 0.1 | 0.1 | 0.2 | 0.1 | x |
|  | 2 | 1 | 1 | 1 | 1 | 2 | 2 | 1 | 1 | 1 | 1 | 2 | 1 |  |
| Emodin (mass %) | 29.1 | 3.4 | 9.8 | 1.7 | 0.1 | 2.3 | 0.5 | 1.8 | 1.5 | 0.2 | 5.8 | 3.8 | 3.0 | x |
|  | 2 | 1 | 1 | 1 | 1 | 2 | 2 | 1 | 1 | 1 | 1 | 2 | 1 |  |
| Root infection rate M (%) | 12.2 | x | 0.8 | 5.0 | x | 0.8 | 4.0 | x | x | 0.1 | x | 0.1 | x | x |
|  | 3 |  | 1 | 1 |  | 3 | 3 |  |  | 1 |  | 3 |  |  |
| Root infection rate F (%) | 12.7 | x | 0.1 | 4.2 | x | 0.3 | 2.2 | x | x | 0.6 | x | 1.2 | x | x |
|  | 3 |  | 1 | 1 |  | 3 | 3 |  |  | 1 |  | 3 |  |  |

Table 3. Plant characteristics measured and tested in 2007. Results of four-way ANOVA (F-values and degrees of freedom) with the following factors: CLONE = knotweed clone; INOC = mycorrhizal inoculation; N = nitrogen level; LF DMG = leaf damage. x = non-tested.
